# Microarray analysis of verbenalin-treated human amniotic epithelial cells reveals therapeutic potential for Alzheimer’s Disease

**DOI:** 10.18632/aging.102985

**Published:** 2020-03-29

**Authors:** Farhana Ferdousi, Shinji Kondo, Kazunori Sasaki, Yoshiaki Uchida, Nobuhiro Ohkohchi, Yun-Wen Zheng, Hiroko Isoda

**Affiliations:** 1 Alliance for Research on the Mediterranean and North Africa (ARENA), University of Tsukuba, Tsukuba 305-8577, Ibaraki, Japan; 2 R&D Center for Tailor-Made QOL, University of Tsukuba, Tsukuba 305-8550, Ibaraki, Japan; 3 National Institute of Advanced Industrial Science and Technology (AIST), Tsukuba 305-8565, Ibaraki, Japan; 4 School of Integrative and Global Majors, University of Tsukuba, Tsukuba 305-8575, Ibaraki, Japan; 5 Department of Gastrointestinal and Hepato-Biliary-Pancreatic Surgery, Faculty of Medicine, University of Tsukuba, Tsukuba 305-8575, Ibaraki, Japan; 6 Faculty of Life and Environmental Sciences, University of Tsukuba, Tsukuba 305-8575, Ibaraki, Japan

**Keywords:** Alzheimer’s disease, verbenalin, human amnion epithelial cell, microarray analysis, natural compound

## Abstract

Alzheimer’s disease (AD) has become a major world health problem as the population ages. There is still no available treatment that can stop or reverse the progression of AD. Human amnion epithelial cells (hAECs), an alternative source for stem cells, have shown neuroprotective and neurorestorative potentials when transplanted *in vivo*. Besides, studies have suggested that stem cell priming with plant-derived bioactive compounds can enhance stem cell proliferation and differentiation and improve the disease-treating capability of stem cells. Verbenalin is an iridoid glucoside found in medicinal herbs of Verbenaceae family. In the present study, we have conducted microarray gene expression profiling of verbenalin-treated hAECs to explore its therapeutic potential for AD. Gene set enrichment analysis revealed verbenalin treatment significantly enriched AD-associated gene sets. Genes associated with lysosomal dysfunction, pathologic angiogenesis, pathologic protein aggregation, circadian rhythm, age-related neurometabolism, and neurogenesis were differentially expressed in the verbenalin-treated hAECs compared to control cells. Additionally, the neuroprotective effect of verbenalin was confirmed against amyloid beta-induced neurotoxicity in human neuroblastoma SH-SY5Y cells. Our present study is the first to report the therapeutic potential of verbenalin for AD; however, further in-depth research in the *in vitro* and *in vivo* models are required to confirm our preliminary findings.

## INTRODUCTION

Alzheimer’s disease (AD) is a prevalent neurodegenerative disorder accounting for at least two-thirds of cases of dementia in people aged 65 and over. AD is characterized by progressive deterioration of cognitive function and memory [1]. Although increasing age is the most important known risk factor for AD, a combination of genetic, lifestyle, and environmental factors also contribute to the pathologic progress of AD [2]. With a rapidly aging world population, AD has become a major health problem in both developed and developing nations. Globally in 2016, the prevalence of AD and other dementias was estimated to be 43.8 million, with 2.4 million deaths making AD and dementia the fifth-largest cause of death. It is projected that by 2050, the number of people living with AD and dementia would be over 100 million [3]. Currently, cholinesterase inhibitors and memantine are the only medicines approved in the US and Europe; however, they only provide short-term improvement of AD symptoms for a short period of six to eighteen months. There are more than 100 compounds under investigation for the possible treatment of AD [4]. Continuing efforts are still required to develop medicines as well as novel, practical strategies that would slow the progression, halt, or prevent AD and other dementias and recover cognitive functions.

Current advances in stem-cell-based therapies or approaches, such as the promotion of endogenous neurogenesis, transplantation of exogenous stem cells, etc. have shed light on novel treatment strategies of AD [5, 6]. However, the high cost, time-consuming, and labor-intensive nature of stem cell therapy limit its use. And therefore, identification of a suitable stem cell source with therapeutic applications has become a top priority. Human amnion epithelial cells (hAECs), isolated from a medical waste product such as discarded term placenta, are gaining interest as a new alternative source of stem cells as they have similar pluripotent and multipotent properties of stem cells. Moreover, they have the further advantage over embryonic stem cells (ESCs) and induced pluripotent stem cells (iPSCs), such as they are readily available, they do not form teratomas *in vivo*, have low immunogenicity and low rejection rate, have immunomodulatory and anti-inflammatory properties, and pose far fewer ethical concerns [7]. Evidences have shown that hAECs can cross the blood-brain barrier where they can engraft and survive for up to 60 days, and eventually can promote the survival and regeneration of neurons, synthesize and release neurotrophic factors and neurotransmitters, and reestablish the damaged neural connections, suggesting that hAECs may be one of the most promising candidates for cell-based therapy of neurological diseases [8–11].

Recent advanced researches on plant extracts and their bioactive compounds are bringing into light their importance in regenerative medicine. In this regard, several priming approaches using natural compounds have been proposed in recent years to activate stem cells for proliferation and differentiation and to improve the survival, function, and therapeutic efficacy of stem cells [12–14]. Moreover, recent evidence shows that the outcome of stem cell therapy in neurodegenerative diseases can be improved through the combination of adjunct treatments [15, 16]. Natural compounds of dietary origin, known as nutraceuticals, would be promising candidates to produce synergistic effects with stem cell therapy. Several studies have already suggested the protective properties of natural compounds against age-related neurodegenerative diseases [17, 18]. Therefore, *in vitro* enrichment or preconditioning of stem cells in the presence of a specific plant extract or its pharmacologically active substance can open a new horizon for regenerative medicine and treatment; however, exploration of the strategies in this regard has been sparse.

Verbenalin is an iridoid glucoside found in medicinal herb *Verbena officinalis* (*V. officinalis*) and other plants of the Verbenaceae family, such as *Lippia citriodora* [19–21]. The plant *V. officinalis*, also known as “holy plant”, is native to Europe and the Mediterranean region. Herbal tea made from *V. officinalis* has traditionally been used for the treatment of insomnia as well as a home remedy for headache, fever, depression, and nervous exhaustion. Verbenalin, one of the major constituents of this plant, has been reported to exhibit sleep-promoting and antioxidant activities [20, 22, 23]. In our previous study, we have reported relaxation and anti-depressant effects of lemon verbena (*Lippia citriodora*) extract, rich in verbascoside and verbenalin, both in the *in vitro* and *in vivo* models [24]. In the present study, we have treated hAECs with verbenalin for seven days and conducted microarray analysis to investigate the changes in gene expression and to explore its therapeutic potential for AD. Additionally, we evaluated the neuroprotective effect of verbenalin against amyloid beta-induced neurotoxicity in human neuroblastoma SH-SY5Y cells.

## RESULTS

### Characteristics of differentially expressed genes (DEGs)

In the present study, control hAEC spheroids were maintained in the placental basal medium, and the treatment hAEC spheroids were treated with 20 μM of verbenalin for seven days. The effective concentration of verbenalin on hAEC was determined using the mitochondrial-dependent reduction of 3-(4,5-dimethylthiazol-2- yl)-2,5-diphenyl tetrazolium bromide (MTT) assay (Supplementary Figure 1). Microarray analysis was conducted on three biological replicates of day 7 (d7) control and treatment samples, and two biological replicates of day 0 (d0) control samples. Genes satisfy both *p*-value <0.05 (one-way between-subjects ANOVA) and fold-change (in linear space) > 1.1 criteria simultaneously were considered as differentially expressed genes (DEGs) and were included for gene ontology (GO) analysis.

**Figure 1:**
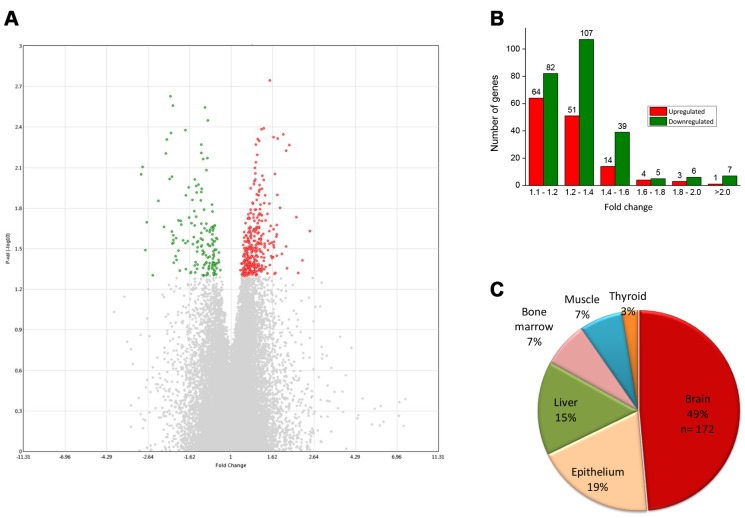
(**A**) Volcano plot displaying DEGs between verbenalin-treated and untreated-control hAECs on day 7 (performed in Transcriptome Analysis Console version 4 software). The vertical axis (y-axis) corresponds to -log10 p-value of the ANOVA p-values, and the horizontal axis (x-axis) displays linear fold change. The red dots represent the up-regulated genes; the green dots represent the downregulated genes. (**B**) Distribution of fold changes in mRNA expression levels in verbenalin-treated hAECs (**C**) Pie chart showing the enriched (p < 0.05) tissue expressions by the DEGs between verbenalin-treated and untreated-control hAECs on day 7 (analyzed by DAVID online tool).

We found a total of 383 unique genes were consistently differentially expressed in all three replicates of verbenalin-treated cells and were considered as DEGs for further analysis. Among the DEGs, 137 genes were upregulated, and 246 genes were downregulated. Figure 1A shows a volcano plot displaying the DEGs. The red dots represent the significantly upregulated genes, and the green dots represent the significantly downregulated genes. There were several genes which showed large relative differences, or fold changes, however, the fold changes were not consistent among the replicates, thus, were not included for further analysis (Figure 1A, grey dots represent the non-significant genes).

Figure 1B shows the distribution of fold changes of the DEGs. Most of the DEGs (80%) showed fold change <1.4, probably because we did not add any supplement other than verbenalin in the treated cells. For the GO analysis, we have included all the genes with fold change >1.1 (and, p<0.05) to explore the molecular changes which might be small in magnitude but are consistent. Tissue expression analysis by the ‘functional annotation’ tool of DAVID (Database for Annotation, Visualization and Integrated Discovery) software revealed that 49% of DEGs were brain-specific (Figure 1C). Further analysis of brain-specific DEGs showed that 34% genes were previously reported to be expressed in pons (n= 131), 23% in medulla oblongata (n= 90), 21% in parietal lobe (n= 82), and 20% in subthalamic nucleus (n= 75). Gene family analysis of the DEGs revealed 25 genes were transcription factors (TFs), 11 were protein kinases, and seven were growth factors. Top 20 significantly upregulated and downregulated genes and their related GO have been listed in Supplementary Tables 1 and 2, respectively.

### Significantly enriched cellular components and biological process

**Figure 2:**
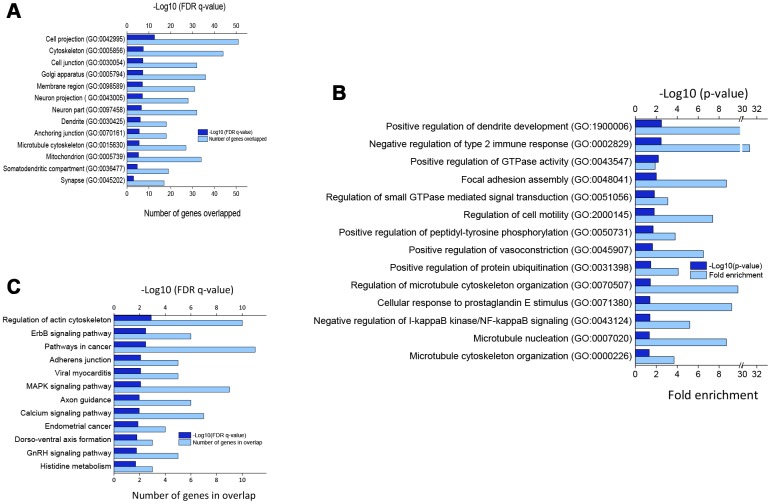
(**A**) Significantly enriched cellular components for DEGs. (**B**) Top biological processes as per p-value (modified Fisher’s exact) by DEGs. (**C**) Significantly enriched KEGG pathways by DEGs (p < 0.05; modified Fisher’s exact test). All the gene ontology enrichment analyses were performed using DAVID online tool.

Figure 2 shows significantly enriched cellular components (Figure 2A) and biological processes (Figure 2B) by DEGs in verbenalin-treated hAECs according to false discovery rate (FDR) q-value and p-value, respectively. Significantly enriched top cellular components include, but not limited to, cell projection (GO: 0042995), cytoskeleton (GO: 0005856), cell junction (GO: 0030054), neuron part (GO: 0097458), dendrite (GO: 0030425), and synapse (GO: 0045202). Significantly enriched top biological processes are positive regulation of dendrite development (GO: 1900006), negative regulation of type 2 immune response (GO: 0002829), guanosine triphosphatase (GTPase) activity (GO: 0043547), vasoconstriction (GO: 0045907), protein ubiquitination (GO: 0031398), regulation of GTPase mediated signal transduction (GO: 0051056), cell motility (GO: 20000145), and microtubule cytoskeleton organization (GO: 0000226).

### Significantly enriched Kyoto Encyclopedia of Genes and Genomes (KEGG) pathways

Figure 2C shows the significantly enriched (p < 0.05; modified Fisher’s exact test) KEGG pathways by the DEGs. Several signaling pathways, namely ErbB, mitogen-activated protein kinase (MAPK), Gonadotropin-releasing hormone (GnRH), and calcium signaling pathways, as well as pathways in cancer, were significantly overrepresented. Other significantly enriched signaling pathways include regulation of actin cytoskeleton, adherens junction, axon guidance, and dorsoventral axis.

### Gene set enrichment analysis (GSEA) reveals regulation of AD-related gene sets

Table 1 listed important gene sets that were significantly enriched by the DEGs between d7 verbenalin-treated and control hAECs. Gene sets were identified using the Molecular Signatures Database (MSigDB) of GSEA. Interestingly, we found that 44 DEGs were overlapped with the gene set that is upregulated in the brain from patients with AD (p=1.31 e^-12^). We also found that 29 DEGs were overlapped with the gene set that is downregulated in the brain from AD patients (p=1.02 e^-7^) [25]. Neurogenesis-, neuron differentiation-, and nervous system development- associated gene sets were also highly enriched.

**Table 1:** Significantly enriched gene sets by DEGs between verbenalin-treated and control cells.

Gene set^*^	**Systematic name, Gene Ontology**	**No. of genes in set**	**No. of Genes in Overlap**	**p-value**	**FDR q-value**
Genes up-regulated in brain from patients with Alzheimer’s disease	M12921	1691	44	1.31 e^-12^	2.87 e^-9^
Neurogenesis^1^	M13908, GO:0022008	1402	36	2.42 e^-10^	1.87 e^-7^
Regulation of neuron differentiation^2^	M12739, GO:0045664	554	19	6.44 e^-8^	1.25 e^-5^
Regulation of nervous system development^3^	M11450, GO:0051960	750	22	9.09 e^-8^	1.65 e^-5^
Genes down-regulated in brain from patients with Alzheimer’s disease	M17728	1237	29	1.02 e-7	2.99 e^-5^
Table 1. Significantly enriched gene sets by DEGs between verbenalin-treated and control cellsGenes up-regulated during later stage of differentiation of Oli-Neu cells (oligodendroglial precursor)	M2368,	570	9	1.64 e^-5^	1.17 e^-2^
Neuromuscular process^4^	M15744, GO:0050905	97	4	1.15 e^-4^	2.84 e^-2^

^*^GSEA online software (http://software.broadinstitute.org/gsea/index.jsp).

^1^Generation of cells within the nervous system.

^2^Any process that modulates the frequency, rate or extent of neuron differentiation.

^3^Any process that modulates the frequency, rate or extent of nervous system development, the origin and formation of nervous tissue.

^4^Any process pertaining to the functions of the nervous and muscular systems of an organism.

### Verbenalin treatment regulated AD-associated genes in hAECs

**Figure 3:**
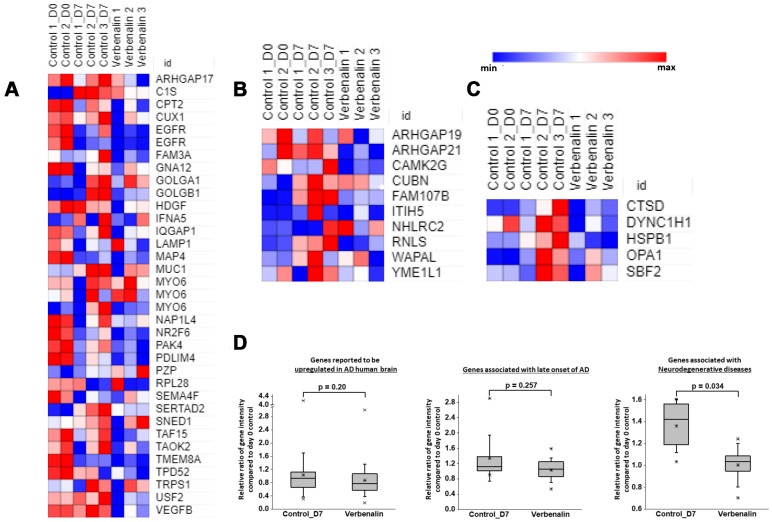
Heat maps showing relative expression intensity of genes reported to be (**A**) upregulated in AD human brain, (**B**) strongly associated with late-onset of AD, (**C**) associated with neurodegenerative diseases in untreated control hAECs on day 0 and day 7, and in verbenalin-treated hAECs on day 7. (**D**) Boxplots for the relative ratios of gene intensity (genes presented in the heat maps) in day 7 control (Control_D7) and verbenalin-treated hAECs compared with day 0 control. Box ranges from 25^th^ to 75^th^ percentile, the line in the middle represents the median value, the whiskers represent the min, max, and mean values, and the error bar represents the SD. Significance was computed by One-way ANOVA for linear distribution and Mann-Whitney U test for nonlinear distribution. Heat maps were generated using Morpheus online tool.

We found that a total of 73 AD-associated genes were regulated in the verbenalin-treated hAECs, among which 44 were reported to be upregulated (termed as ‘group A’ genes), whereas 29 genes were reported to be downregulated (termed as ‘group B’ genes) in the brain from patients with AD [25]. In the verbenalin-treated hAECs, 32 ‘group A’ genes were downregulated, and 12 were upregulated. Figure 3A shows the heatmap of the relative gene intensity of the downregulated AD-associated genes. Seven genes of this ‘group A’ genes were previously reported to be upregulated at the incipient stage of AD, namely TAO Kinase 2 (*TAOK2*), Carnitine Palmitoyl Transferase 2 (*CPT2*), Tumor Protein D52 (*TPD52*), Interferon, Alpha 5 (*IFNA5*), Transmembrane Protein 8A (*TMEM8A*), Family with Sequence Similarity 3, Member A (*FAM3A*), and Rho GTPase Activating Protein (*ARHGAP*) 17 (*ARHGAP 17*), all of which were downregulated in the treatment cells. Several transcription factors were among the ‘group A’ genes, such as Cut-Like Homeobox 1 (*CUX1*), Nuclear Receptor Subfamily 2, Group F, Member 6 (*NR2F6*), SERTA Domain Containing 2 (*SERTAD2*), TATA-Box Binding Protein Associated Factor (*TAF)* 11 (*TAF11*), *TAF15*, Transcription Factor 7-Like 1 (*TCF7L1*), Trichorhinophalangeal Syndrome I (*TRPS1*), and Upstream Transcription Factor 2 (*USF2*). Among the ‘group B’ genes, 25 were downregulated, and four were upregulated in the verbenalin-treated cells. Further analysis of these 25 genes showed overlap with the genes associated with ‘cell death’ (n=6), ‘genes whose expression significantly and positively correlated with the density of calbindin-containing gamma-Aminobutyric acid-ergic (GABAergic) neurons from prefrontal cortex (Brodmann area 9 (BA9) brain region) across all subjects with psychiatric disorders, such as bipolar disorder, depression, and schizophrenia’ (n=10) [26], and ‘genes whose expression significantly and positively correlated with oligodendrocyte density in layer VI (n=7), and layer III (n=5) of the BA9 brain region in patients with bipolar disorder’ [26]. We also found 11 genes of ‘group B’ were reported to be strongly associated with late-onset of AD [27] (Figure 3B), and five genes were associated with neurodegeneration (Figure 3C). Figure 3D shows the boxplots for the relative ratios of gene intensity (genes presented in the heat maps) compared between d7 control *vs*. d0 control and verbenalin-treated hAECs *vs*. d0 control. When compared with d0 control, d7 verbenalin-treated hAECs showed a better effect on AD-associated genes than d7 untreated control hAECS. Several genes (n = 22) associated with interactions of pathological hallmark proteins tubulin polymerization promoting protein/P25, β-amyloid, and α-synuclein [28] were also found to be regulated in the treatment cells. List of AD-associated genes and their fold changes compared with d0 and d7 controls is given in the Supplementary Table 3.

### Verbenalin treatment regulated the gene expression of the receptors of the ErbB pathway

**Figure 4:**
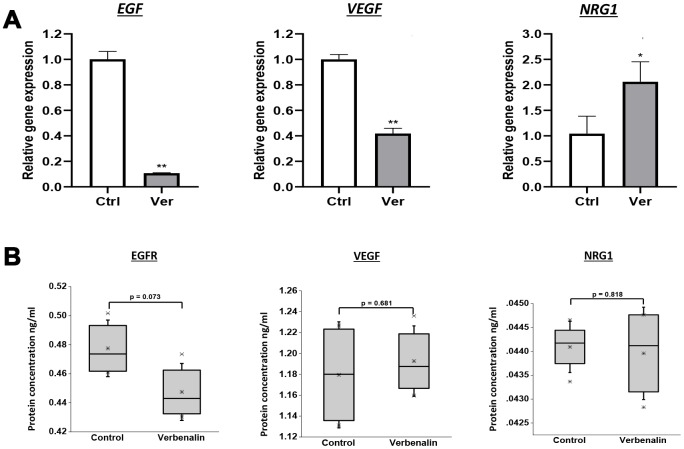
**Effect of verbenalin treatment on the expressions of EGF, VEGF, and NRG1.** The hAECs were treated with 20 μM of verbenalin (Ver) for 7 days, while the control cells were maintained in the placental basal medium. (**A**) Gene expressions were evaluated by real-time PCR. Each value represents the mean ± SD (n = 3). Asterisks refer to statistical significance (*p < 0.05, **p < 0.01) by One-way ANOVA as compared with control (Ctrl). (**B**) Boxplots of protein concentration (ng/ml) obtained by ELISA (n = 4). Box ranges from 25^th^ to 75^th^ percentile, the line in the middle represents the median value, the whiskers represent the min, max, and mean values, and the error bar represents the SD. The difference in protein concentration between treatment and control group was measured using One-way ANOVA for linear distribution.

The dysregulation of ErbB signaling in humans is associated with the development of AD. In the microarray analysis, we found that verbenalin treatment significantly downregulated the expression of epidermal growth factor (EGF) receptor (*EGFR*) (fold change -1.41), and vascular endothelial growth factor B (*VEGFB*) (fold change -1.89). On the other hand, the expression of neuregulin 1 (*NRG1*) was significantly upregulated (fold change 1.34). A similar gene expression result was found in the real-time PCR (RT-PCR) analysis (Figure 4A). Although not significant, EGFR and NRG1 showed similar protein expression patterns as gene expression (Figure 4B). However, VEGF showed a slight upregulation in protein expression (Figure 4B). *NRG1* is the first discovered member of the NRG family that contains the EGF-like domain. NRGs and related EGF-domain containing proteins interact with different receptor tyrosine kinases of the ERBB family (ERBB1- 4) and initiate intracellular signaling pathways in a specific way. NRG1 is the direct ligand for ERBB3 and ERBB4 tyrosine kinase receptors, and concomitantly recruits ERBB1 and ERBB2 coreceptors, resulting in ligand-stimulated tyrosine phosphorylation and activation of the ERBB receptors. Adenomatous polyposis coli (*APC*) that acts as a mediator of ERBB2-dependent stabilization of microtubules at the cell cortex was downregulated (fold change -1.13).

### Verbenalin treatment regulated expression of genes of Rho family GTPases

The Rho GTPases belong to the Ras superfamily of small (molecular weight ~21kDa) guanine nucleotide-binding proteins (G-proteins). The most extensively studied members of the Rho family are Ras Homolog Family Member A (*RHOA*), Ras-related C3 botulinum toxin substrate 1 (*RAC1*), and cell division cycle 42 (*CDC42*). We found that verbenalin treatment significantly downregulated the expression of several genes of ARHGAP, such as *ARHGAP21* (fold change -2.00), *ARHGAP17* (fold change -1.38), *ARHGAP4* (fold change -1.37)*, ARHGAP19* (fold change -1.11). ARHGAP21 functions as the GTPase activator for *RHOA* and *CDC42*, ARHGAP17 for *CDC42*, and ARHGAP19 for *RHOA*. We also found the downregulation of family with sequence similarity 65, member B (*FAM65B*), an inhibitor of the small GTPase RhoA (fold change - 1.93). Additionally, pleckstrin homology domain-containing family G member 6 (*PLEKHG6*), a guanine nucleotide exchange factor activating the small GTPase RhoA, was also downregulated (fold change -1.38).

### Verbenalin treatment regulated genes associated with circadian entrainment

Circadian entrainment is the biological process by which endogenous oscillations are synchronized with external cues, such as daily light and temperature cycles, within a period of ~24 h. The circadian clock coordinates the daily molecular, hormonal, physiological, and behavioral rhythms. We found several genes associated with circadian rhythm were downregulated in the verbenalin-treated hAECs, such as TFs NK2 homeobox 1 (*NKX2-1*; fold change -1.13) and retinoic acid-induced protein 1 (*RAI1*; fold change -1.13), imprinted gene GNAS complex locus (*GNAS*; fold change -1.27), and G protein beta polypeptide 1 (*GNB1*; fold change -1.19). Although not specific, there was also the downregulation of voltage-gated Ca^2+^ channel *CACNA1C* (fold change -1.13) and Ca^2+^/calmodulin-dependent kinase (CAMK) II gamma (*CAMK2G*; fold change -2.07) in the verbenalin-treated cells.

### Verbenalin treatment showed neuroprotective effects against Aβ-induced cytotoxicity in human neuroblastoma SH-SY5Y cells

**Figure 5:**
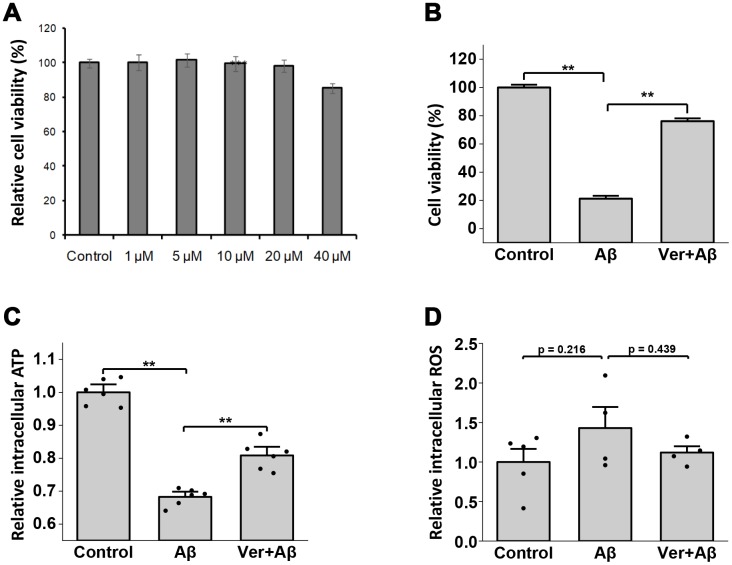
**Neuroprotective effects of verbenalin (Ver) on amyloid beta (Aβ)-induced toxicity in human neuroblastoma SH-SY5Y cells.** (**A**) Cells were exposed to verbenalin at concentrations of 1, 5, 10, 20, and 40 μM for 72 h. The control cells were not treated. Cell viability was measured by the MTT assay and was calculated as a percentage of that in the control group (100%). The results are expressed as the means ± standard error of the mean (SEM) of independent experiments (n = 6, 96-well plate). ***p < 0.001 as compared to control. Cells were pre-treated with 20 μM verbenalin for 24 h and then exposed to 5 μM Aβ for 72 h. The results are expressed as the means ± standard error of the mean (SEM) of independent experiments (n = 6, 96-well plate). ^†^p < 0.1, *p < 0.05, **p < 0.01 compared with the group exposed to Aβ only (ANOVA followed by Dunnett’s multiple comparisons test). (**B**) Cell viability was measured by the MTT assay and was calculated as a percentage of that in the control group (100%). (**C**) A bioluminescence assay was used to measure cellular ATP levels, and the results are shown as relative intracellular ATP levels. (**D**) Levels of intracellular reactive oxygen species (ROS) were measured using a fluorescence cell-based assay, and results are shown as relative intracellular ROS (n=4).

As unexpectedly, our study results showed that verbenalin treatment could significantly regulate AD-associated genes in hAECs, we investigated its neuroprotective effects against Aβ-induced cytotoxicity in human neuroblastoma-derived SH-SY5Y cells. The human SH-SY5Y cell line is a widely-used cellular model to examine the toxic effects of amyloid peptides. We found that verbenalin was nontoxic up to the concentration of 20 μM (Figure 5A). Therefore, 20 μM of verbenalin was used for determining its effects on Aβ-induced neuronal cell damage. When SH-SY5Y cells were exposed to 5 μM of Aβ for 72 h, there was significant cell death compared with untreated controls (Figure 5B). However, in cultures pre-treated with 20 μM of verbenalin for 24 h (Figure 5B), the Aβ-induced cell death was significantly reduced compared to only Aβ-treated conditions, suggesting the neuroprotective effect of verbenalin against Aβ-induced cytotoxicity in SH-SY5Y cells.

### Verbenalin treatment significantly ameliorated the Aβ-induced decline of ATP levels in SH-SY5Y cells

Figure 5C shows the effect of verbenalin treatment on Aβ-induced ATP decline. Exposure to 5 μM of Aβ for 24 h resulted in a significant decrease in ATP production compared to control cells. However, pretreatment with verbenalin for 24 h could rescue the reduction of ATP production in Aβ-treated SH-SY5Y cells (p < 0.01).

### Verbenalin treatment attenuated Aβ-induced reactive oxygen species (ROS) generation in SH-SY5Y cells

The effect of verbenalin treatment on oxidative stress was detected by testing the level of intracellular ROS. Figure 5D shows that after exposure to 5 μM of Aβ for 24 h, ROS production was increased compared to untreated cells (p = 0.22). When the cells were pretreated with 20 μM of verbenalin for 24 h, ROS production was decreased compared to only Aβ-treated SH-SY5Y Cells (p = 0.44), suggesting the preventive effect of verbenalin against Aβ-induced oxidative stress. However, the changes did not achieve statistical significance.

### Verbenalin treatment regulated the expressions of *EGFR*, *VEGF,* and *NRG1* in Aβ-induced SH-SY5Y cells

**Figure 6:**
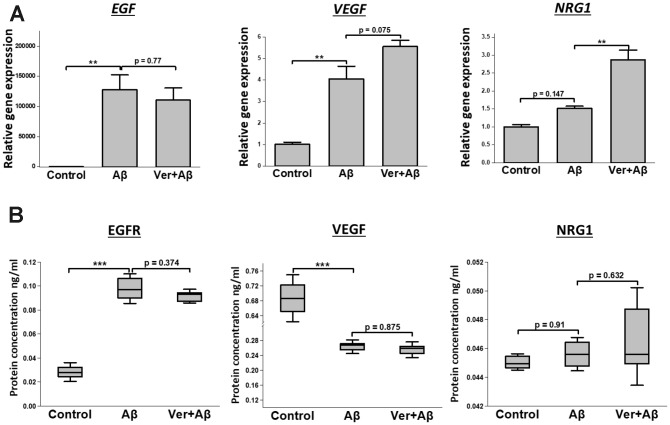
**Effect of verbenalin treatment on the expressions of EGF, VEGF, and NRG1 in Aβ-induced human neuroblastoma SH-SY5Y cells.** (**A**) Gene expressions were evaluated by real-time PCR. Each value represents the mean ± SD (n = 4). (**B**) Boxplots of protein concentration (ng/ml) obtained by ELISA (n = 4). Box ranges from 25^th^ to 75^th^ percentile; the line in the middle represents the median value; the error bar represents the SD. Asterisks refer to statistical significance (*p < 0.05, **p < 0.01, ***p < 0.001) by One-way ANOVA followed by Dunnett’s multiple comparisons test (for linear distribution) as compared with only Aβ-treated group.

We investigated how verbenalin treatment could modulate the expressions of *EGFR*, *VEGF,* and *NRG1* in Aβ-induced neurotoxic condition. Figure 6A shows that verbenalin could inhibit Aβ-induced *EGFR* activation, and upregulated the expressions of *VEGF* and *NRG1*. EGFR and NRG1 showed similar protein expression patterns as gene expression (Figure 6B).

## DISCUSSION

AD is a progressive neurodegenerative disease overlaid with neuropsychiatric and behavioral symptoms [29]. The current pharmacologic therapy for AD only provides short term alleviation of symptoms. In recent years, hAECs and alternative sources of adult stem cells have been gaining interest in regenerative medicine for the treatment of neurodegenerative diseases [10, 30]. Xue et al., have reported that intracerebroventricular transplantation of hAEC in the transgenic AD model mice could improve cognitive functions, and increase acetylcholine levels and the number of hippocampal neurites [8]. Another study has reported that intravenous injection of human amniotic membrane-derived mesenchymal stem cells in transgenic AD mouse models improved AD pathology and memory function through regulating oxidative stress [31]. With advances in stem cell biology and regenerative medicine, it is reasonable to construct new approaches that may improve the treatment options. Recently, plant extracts and their bioactive compounds have received considerable attention because of their distinct pharmacology profiles, such as the rapid onset of action, less side effect profile, potential drug synergies, and most importantly because of their ability to improve proliferation, differentiation and therapeutic efficacy of stem cells [2, 4, 12–14]. On the other hand, microarray gene expression profiling is a useful tool to explore genome-wide expression patterns that are activated during studied biological conditions and provides a foundation for further examination of molecular mechanisms and regulatory pathways. Therefore, in the present study, we conducted a microarray analysis of the gene expression pattern of verbenalin-treated hAECs to explore its health beneficial potentials.

The study results revealed that verbenalin treatment could significantly enrich the priori-defined AD-associated gene sets (Table 1) that analyzed hippocampal gene expression of AD patients of varying severity [25]. Most importantly, we found that verbenalin treatment could significantly downregulate the expression of *EGFR*, which is thought to play the central role in the neuronal and metabolic interaction during the aging process. Several studies have demonstrated the role of EGFR in neurometabolic pathophysiology, aging-related metabolic activity, as well as age-related neuronal survival and regeneration [32, 33]. On the other hand, there was significant upregulation of *NRG1*, a member of growth and differentiation factor containing EGF-like signaling domain. NRG1 is reported to attenuate cognitive function impairments in mice model of AD via inducing neurogenesis [34]. Importantly, NRG1 and other EGF-like proteins interact with receptor tyrosine kinases of the ERBB family and initiate specific intracellular signaling pathways [35]. We also found significant downregulation of several ARHGAPs in verbenalin-treated hAECs, which may contribute to inhibition of tyrosine kinases [36], resulting in inhibition of EGFR. Therefore, the NRG1/ErbB and EGFR/ErbB signaling pathways need to be carefully investigated to confirm the AD-preventing potential of verbenalin in hAECs.

Additionally, VEGFB, the growth factor for endothelial cells, was downregulated in verbenalin-treated hAECs. VEGF is the central component of pathological blood vessel formation, and it was reported that EGFR and ERBB2 signaling pathway plays an essential role in VEGF regulation in carcinoma cells [37]. Postmortem studies on human brains found evidence of increased angiogenesis in the hippocampus, mid-frontal cortex, and other parts of AD brains compared to healthy individuals [38]. The amalgamation of accumulated Aβ and neuroinflammation causes diminished blood perfusion of the brain, leading to hypoperfusion/hypoxia-induced angiogenesis through the upregulation of several pro-angiogenic factors, particularly VEGF [39, 40].

Another important TF that was downregulated in verbenalin-treated hAECs is *NR2F6* (fold change - 1.54), also known as eosinophil cationic protein 2 (*EAR2*). Ear2 deletion was reported to cause early memory and learning deficits in APP/PS1 mice through degeneration of locus ceruleus (LC) and noradrenaline deficiency in AD [41]. It has also been reported that abnormal development of LC in *Ear2* deficient mice leads to impaired forebrain clock and affects circadian rhythm [42]. Along with *NR2F6*, we found several other genes of circadian entrainment were up/downregulated in the verbenalin-treated hAECs, such as *GNAS*, *GNB1*, *NKX2-1*, and *RAI1*. It has been reported that loss of imprinting of *Gnas* leads to enhancement of nonrapid eye movement (NREM) and complex cognitive processes, and inhibition of rapid eye movement (REM) and REM–linked behaviors [43]. *GNB1* is a G-protein that is differentially expressed on a night/day basis in the pineal gland [44]. The pineal gland plays a vital role in vertebrate chronobiology by converting time into a hormonal signal and melatonin. *NKX2-1* is a TF that activates the transcription of the GnRH receptor and plays a role in enhancing the circadian oscillation [45, 46]. *RAI1* is the transcriptional regulator of the circadian clock components. It positively regulates the transcriptional activity of CLOCK, a core component of the circadian clock, through chromatin remodeling by interacting with other proteins in chromatin as well as proteins in the basic transcriptional machinery [47]. Previous studies have already reported the sleep-promoting effect of verbenalin [22]. Synchronizing circadian rhythms may be an inexpensive way to promote healthy aging and delay the onset of neurodegenerative diseases such as AD [48, 49].

Other top downregulated AD-associated genes include *TMEM8A*, *USF2*, PDZ and LIM domain 4 (*PDLIM4*), and *TAF15* (Supplementary Table 3). Lysosomal protein TMEM8A is a hallmark for lysosomal dysfunction and is associated with recessive inherited lysosomal storage disorders [50]. However, there is increasing evidence that lysosomes play a central role in the pathogenesis of common neurodegenerative diseases [51–53]. Nixon et al. identified cathepsins in amyloid-β plaques, confirming the broad dysfunction of the lysosomal system in AD [51]. In our study, we found that verbenalin treatment could significantly downregulate the expression of Cathepsin D (*CTSD*; fold change -1.51), a suggested therapeutic target for AD [54]. USFs are essential genes that tie cholesterol metabolism and AD together. USFs regulate genes associated with synaptic plasticity, neuronal survival, and differentiation. Additionally, Isotalo et al. reported an association between USF1 and AD-related lesions [55]. USF1 regulates lipid metabolism genes, including apolipoprotein E (*APOE*) and amyloid precursor protein (*APP*). *APOE* and *APP* are the most commonly accepted risk genes for early onset of AD, suggesting the involvement of lipid metabolism disorder in AD progression.

TAF15 is an RNA binding protein (RBP) and is reported to colocalize with tau pathology in neurodegenerative diseases [56, 57]. We also found downregulation of heat shock protein gene (heat shock 27kDa protein 1; fold change -1.31) in the verbenalin-treated cells, which is also reported to be associated with pathologic protein aggregation in neurodegenerative diseases [56]. Additionally, significantly upregulated annotation terms by the DEGs in verbenalin-treated hAECS include ‘positive regulation of protein localization (GO: 1904951)’, and ‘positive regulation of protein metabolic process (GO: 0051247)’. Significantly downregulated annotation terms include ‘protein complex binding (GO: 0032403, number of DEGs = 22)’, ‘ribonucleotide binding (GO: 0032553, number of DEGs = 30)’, and ‘cytoskeletal protein binding (GO: 0008092, number of DEGs = 16)’. These findings suggest the regulation of RBPs in verbenalin-treated cells, which may affect pathologic protein aggregation.

Heat maps for relative expression intensity (Figure 3A, 3B, 3C) shows that several AD-associated genes were upregulated in d7 control hAECs compared to d0 control hAECs. Verbenalin treatment could significantly downregulate those AD-associated gene expressions in hAECs. The expression intensities of the genes associated with AD in both verbenalin-treated and untreated (d7 control) hAECs were compared with d0 control hAECs (Figure 3D). The findings suggest that verbenalin treatment had a significant effect on neurodegenerative disease-associated genes compared to untreated hAECs.

As verbenalin treatment could modulate AD-associated genes in hAECs, we further investigated its effect against Aβ-induced neurotoxicity in human neuroblastoma SH-SY5Y cells to confirm its neuroprotective properties (Figures 5 and 6). We found that pretreatment with 20 μM of verbenalin for 24 h could significantly reduce the Aβ-induced cell death (Figure 5B), suggesting the neuroprotective potential of verbenalin. Although the underlying molecular mechanism of AD is still unclear, mitochondrial degeneration and oxidative stress are suggested to be the early triggering factors of AD pathophysiology [58, 59]. Defective mitochondria inhibit the production of ATP and increase the production of ROS. Accumulation of ROS eventually induces oxidative damage. Thus, pharmacological inhibition of ROS generation and activation of ATP production has been considered as feasible therapeutic strategies for AD. We found that verbenalin could significantly ameliorate the Aβ-induced decline of ATP levels (Figure 5C) and attenuate the Aβ-induced ROS generation (Figure 5D) in SH-SY5Y cells. We also investigated how verbenalin treatment could modulate the expressions of *EGFR*, *VEGF*, and *NRG1* in Aβ-induced SH-SY5Y cells. Increased EGFR activity has been linked to the Aβ-induced memory loss, a hallmark of AD progression. Wang et al., have reported that EGFR inhibitors are capable of rescuing the Aβ-induced memory loss in both transgenic fruit fly and transgenic mouse models and have suggested that inhibition of Aβ-induced EGFR activation might be an effective way to treat Aβ-induced memory loss in AD. We found that verbenalin treatment could downregulate Aβ-induced EGFR expression. We also found that verbenalin could significantly upregulate NRG1 expression in Aβ-induced SH-SY5Y cells. NRG1 is widely expressed in the adult human brain [60]. Although it is not clear yet how Aβ aggregation affects NRG1 expression in the AD brain, it is evident that NRG1 has therapeutic potential for AD by inducing neurogenesis, improving cognitive deficits, and restoring synaptic plasticity with [61] or without [63] affecting Aβ level. The angiogenic factor VEGF is implicated in pathological angiogenesis in the AD brain [39, 40]; however, it is reported that Aβ antagonizes VEGF activity both *in vitro* and *in vivo* in a transgenic mouse model of AD [62]. The exogenous addition of VEGF can partially rescue the anti-angiogenic effect of Aβ peptides *in vitro* [62]. Another interesting study by Garcia and colleagues [63] showed that transplantation of VEGF overexpressing bone marrow mesenchymal stem cells in the hippocampus of AD transgenic mice could promote neovascularization, and reduce the number of Aβ plaques. In our study, we found that verbenalin treatment significantly reduced *VEGF* expression in hAECs (Figure 4A) but increased its expression in Aβ-induced SH-SY5Y cells. Therefore, the effect of verbenalin on VEGF requires to be evaluated in *in vivo* condition.

One of the advantages of plant-derived natural compounds is that they contain multiple agents that can target multiple pathologies simultaneously, therefore, are found more efficacious than the traditional drugs when faced with complex disease conditions, such as AD. Our study suggests that verbenalin treatment in hAECs may improve its therapeutic potential to AD through modulating the gene expression related to neurometabolic aging, lysosomal dysfunction, pathological angiogenesis, pathological protein aggregation, and circadian rhythms. We have also found that verbenalin could significantly reduce cell death, ameliorate the decline of ATP levels, inhibit the ROS generation and *EGFR* expression, and upregulate *NRG1* expression in Aβ-induced SH-SY5Y cells. As verbenalin treatment could significantly regulate AD-associated genes in hAECs compared to untreated hAECs, it may provide new treatment modalities for neurodegenerative diseases, such as transplantation of verbenalin-treated hAECs, or combination use of hAEC transplantation/systemic administration and oral administration of verbenalin. Future evidence-studies are required to evaluate the afore-mentioned neuroprotective properties of verbenalin systematically.

In conclusion, given the increase in AD prevalence, diverse studies are needed to explore the therapeutic potentials of nature-derived compounds. Even small measurable differences in cognition, behavior, and functioning may become clinically significant to prevent, halt, or cure progressive neurodegenerative diseases. Pretreatment of hAECs or other adult stem cells in the presence of a certain plant extract or its pharmacologically active substance can open a new horizon in regenerative medicine in AD.

## MATERIALS AND METHODS

### Amnion epithelial cells extraction and culture

The detailed methodology has been explained elsewhere [64]. Briefly, AECs were isolated from the delivered term placenta of the mothers who underwent cesarean delivery. The amnion was separated from the chorion manually and was washed with 200 mL of Hank’s Basic Salt Solution – Calcium and Magnesium Free (CMF-HBSS) and then was cut into smaller pieces using surgical scissors. AECs were maintained in Placenta Epithelial Cell Basal Medium (PromoCell, Cat. # C-26140). The medium was changed every 2-4 days. To subculture AECs, the plates were first washed twice with 10 mL of PBS, and then 3 mL of pre-digestion buffer (pre-warmed to 37°C) was added to the plate. After incubation at 37°C for 5 minutes, five mL of 0.05% (w/v) trypsin-EDTA (pre-warmed to 37°C) was added to the plate and incubated at 37°C for 10 minutes. Five mL of Dulbecco’s Modified Eagle Medium (DMEM) was added to stop the reaction. The cell suspension was then centrifuged at 200 RPM for 4 minutes at 4°C twice. After centrifuge, the supernatant was discarded each time, and the cells were suspended in the placental basal medium.

### Preparation of 3D culture plates, spheroid formation, and compound supplement

We used a 3D culture plate (ElplasiaTM, Kuraray Co., Ltd., Cat # RB 500 400 NA 24) for the study. Lipidure ^TM^ (NOF Corporation, Cat. # CMS206; 400 μL) solution was placed into each well of the 3D plate at the concentration of 50 mg in 10 mL absolute ethanol. After two minutes, the Lipidure ^TM^ solution was aspirated out, and the plate was dried for 3 hours. Then 400 μL of PBS was placed in each well. The plate was centrifuged at 2000 g for 15 minutes at room temperature. The PBS was then discarded, and the wells were washed twice with 400 μL of PBS. The plates were then stored in the cell culture incubator until use.

Spheroids were formed by seeding 1 × 10^6^ AECs in Placenta Basal Epithelial Cell Medium into each well of the 24-well plate. The initial culture was maintained for 24 hours. Control samples for d 0 were collected before adding the treatment. For the treatment samples, the medium was changed with 20 μM of verbenalin (Sigma-Aldrich, Japan) every 48 hours for three times. Control samples were maintained in the Placental medium, which was also changed in every 48 hours. Finally, the treatment and control samples were collected from a one-week culture.

### RNA extraction and quantification

RNA was extracted using Isogen (Nippon Gene Co. Ltd., Toyama, Japan) kit following the manufacturer’s guide. NanoDrop 2000 spectrophotometer (ThermoScientific, Wilmington, DE, USA) was used to quantify the integrity of RNA.

### Affymetrix microarray gene expression

We conducted Affymetrix microarray gene expression profiling using GeneChip® 3’ Expression Arrays and 3’ IVT PLUS Reagent Kit (Affymetrix Inc., Santa Clara, CA, USA). We used 250 ng of total RNA from each sample to generate amplified and biotinylated complementary RNA (cRNA) from poly (A) RNA in a total RNA sample following the users’ manual. Human Genome U219 array strips (HG-U219) were hybridized for 16 hours in a 45°C incubator, washed and stained. Imaging was conducted in the GeneAtlas Fluidics and Imaging Station. Each HG-U219 array strip is comprised of more than 530,000 probes, which cover approximately 36,000 transcripts and variants and represent more than 20,000 unique genes.

### Microarray data processing and analysis

Expression Console Software (provided by the Affymetrix) was used to normalize the raw data following the robust multichip average (RMA) algorithm (http://www.affymetrix.com). Subsequent analysis of the gene expression data was carried out in the freely available Transcriptome Analysis Console (TAC) version 4 (Thermofisher Inc.). In this present study, we have considered both fold changes and variability of gene expression for the identification of DEGs. Raw fold change between two experimental conditions does not take the variance of gene expression among the replicates into account. Therefore, it does not provide statistical confidence that the genes will show similar fold-change threshold in future experiments. Therefore, we defined DEGs as the genes that satisfy both *p*-value <0.05 (one-way between-subjects ANOVA) and fold-change (in linear space) > 1.1 criteria simultaneously. MSigDB of GSEA was used to determine whether a priori-defined set of genes shows statistically significant and concordant differences between two biological states, i.e., verbenalin-treated versus non-treated control (https://software.broadinstitute.org/gsea/index.jsp) [65, 66]. MSigDB emphasizes a genomic, unbiased approach to define the gene sets, which are curated from published expression profiles and allows researchers to evaluate microarray data at the level of gene sets, which tend to be more reproducible and more interpretable. Gene annotation, tissue expression, and pathway analysis for the DEGs were conducted using an online data mining tool DAVID ver. 6.8 [67, 68]. Heat maps were generated using visualization software Morpheus (https://software.broadinstitute.org/morpheus). All data generated or analyzed during this study are included in this published article and its supplementary files. Microarray data are deposited in the Gene Expression Omnibus (GEO) under Accession Number: GSE137061 (https://www.ncbi.nlm.nih.gov/geo/info/linking.html).

### Real-time PCR

To synthesize cDNA from total RNA, the SuperScript III reverse transcriptase kit (Invitrogen, Carlsbad, CA, USA) was used. Primers and probes for human vascular endothelial growth factor B (*VEGFB*) (Hs00173634_m1), human epidermal growth factor receptor (*EGFR*) (Hs01076090_m1), human neuregulin 1 (*NRG1*) (Hs01101538_m1) and human glyceraldehyde-3-phosphate dehydrogenase (*GAPDH*) (Hs02786624_g1) were purchased from Applied Biosystems (Foster City, CA, USA). The gene expression was normalized to *GAPDH* expression. The real-time PCR amplification and product detection were performed with a 7500 Fast Real-time PCR system (Applied Biosystems) using TaqMan Gene Expression Master Mix (Applied Biosystems). Each reaction was run in triplicate, and data were analyzed using the ΔΔCt method.

### Cell cytotoxicity test

The SH-SY5Y cells (American Type Culture Collection (ATCC), Manassas, VA, USA) and hAECs were seeded in 96-well plates at a density of 2.0 × 10^5^ cells/ml and 1.0 × 10^5^ cells/ml respectively and were incubated for 24 h. The following day, the cells were treated with various concentrations (1, 5, 10, 20, and 40 μM) of verbenalin for 72 h. After the treatment, the MTT (Dojindo Laboratories, Kumamoto, Japan) solution was added to each well (10 μl/well) and was incubated at 37°C for 24 h. Then, the generated formazan crystal was dissolved with 100 μl of 10% Sodium Dodecyl Sulfate (SDS) (Nippon Gene, Tokyo, Japan) in each well, and was incubated overnight at 37°C. After that, the absorbance was measured at 570 nm using a microplate reader (Power Scan HT, BioTEK Japan Inc.). The values were normalized to the value of the medium and were calculated as the percentage (%) of control.

### *In vitro* neuroprotection assay

The human neuroblastoma SH-SY5Y cell line was obtained from American Type Culture Collection (ATCC) (Manassas, VA, USA). The cells were maintained in a mixture of 1:1 (v/v) of Dulbecco’s modified eagle medium (DMEM), and nutrient mixture F-12 (Ham) supplemented with 15% heat-inactivated FBS (Gibco, Japan), 1% MEM non-essential amino acids solution (Biological Industries, Beit Haemek, Israel) and 1% penicillin (5000 μg/mL)− streptomycin (5000 IU/mL) solution (Lonza, Basel, Switzerland) on 100 mm culture dish (Falcon, Corning, NY, USA) or 96-well culture plates (Falcon) at 37 °C in a 95% humidified air−5% CO2 incubator. A serum-free Eagle’s minimum essential medium (Opti-MEM) (Gibco, Japan) was used to culture the cells for the neuroprotection assay.

The neuroprotective activity was determined by the MTT reduction assay. SH-SY5Y cells were seeded at a density of 2.0 × 10^4^ cells/well in the 96-well culture plates and incubated for 24 h. The cells were pre-incubated with 20 μM verbenalin in Opti-MEM for 24 h and then were subjected to treatment with 5 μM Aβ and the sample for 24 h. The treated medium was replaced with 100 μL of Opti-MEM in the absence of verbenalin and Aβ. Subsequently, 10 μL of MTT (Dojindo Laboratories, Kumamoto, Japan) dissolved in PBS (−) at 5 mg/mL was added into the medium. After overnight incubation at 37 °C, 100 μL of 10% SDS (w/v) was added and incubated until the formazan product dissolved. The absorbance was measured at the wavelength of 570 nm with a Varioskan Lux multimode microplate reader (Thermo Fisher, Waltham, MA, USA). The proliferation of SH-SY5Y cells was shown by the percentage of the Aβ-treated group.

### ATP assay

SH-SY5Y cells were seeded at a density of 2.0 × 104 cells/well in the 96-well culture plates and incubated for 24 h. The cells were then pre-incubated with 20 μM verbenalin in culture medium for 24 h. After 24 h incubation, the medium was replaced with 100 μl of culture medium containing 5 μM Aβ and 20 μM verbenalin. After 24 h, 100 μl of Cellular ATP measurement reagent (CA2-100, TOYO B-NET, Tokyo, Japan) was added and stirred for 1 min on a plate shaker. Then 150 μl of the suspension was transferred to a white plate and was let stand for 10 min in a luminometer set at 23 °C. The luminescence was measured with a Varioskan Lux multimode microplate reader.

### Cellular ROS assay

Cellular ROS production was measured using OxiSelect Intracellular ROS Assay Kit (Green Fluorescence, STA-342, Cell Biolabs, San Diego, CA, USA) according to the manufacturer’s instructions. Briefly, SH-SY5Y cells were seeded at a density of 2.0 × 10^4^ cells/well in the 96-well culture plates and were incubated for 24 h. The cells were then pre-incubated with 20 μM verbenalin in culture medium for 24 h. The culture medium was later replaced with 100 μl of 0.1x DCFH-DA and the cells were incubated for 1 h at 37 °C. Then 0.1x DCFH-DA was replaced with 100 μl of Opti-MEM containing 5 μM of Aβ and 20 of μM verbenalin. The cells were incubated at 37 °C for 24 h. The sample-containing medium was replaced with 200 μl of 1 × Cell Lysis Buffer, shaken for 1 min on a plate shaker, and incubated for 5 min at room temperature (24 °C). Finally, 150 μl of cell lysate was transferred to a black plate. Fluorescence wavelength was detected at 480nm excitation / 530nm emission with a Varioskan Lux multimode microplate reader.

### Protein isolation and detection

SH-SY5Y cells were seeded at a density of 6.0 × 105 cells / 3ml / well in a 6-well plate. After 24 h of incubation at 37 °C, the cells were pre-incubated in 3 ml of Opti-MEM medium containing 20 μM Verbenalin for another 24 h. The medium was then replaced with 3 ml of Opti-MEM medium containing 5 μM Aβ and 20 μM verbenalin. The medium was collected after 72 h. commercially available enzyme-linked immunosorbent assay (ELISA) kits were used to measure VEGF (ab100662), NRG1 (ab100614), and EGFR (ab100505) according to the manufacturer’s instructions (Abcam, Cambridge, UK). The absorbance was measured at 450 nm on a Varioskan Lux multimode microplate reader.

### Statistical analysis

Data were analyzed using GraphPad Prism (version 8.0, GraphPad Software Inc., San Diego, CA) and SPSS ver.26 (Armonk, NY: IBM Corp). Data were expressed as the means ± standard error of the mean (SEM) unless otherwise mentioned. One-way analysis of variance (ANOVA) followed by Dunnett’s multiple comparisons test for linear distribution and Mann-Whitney U test for nonlinear distribution was carried out for the comparisons between treatment groups. Differences were considered statistically significant at the value of P < 0.05. Graphs were prepared using OriginPro software (OriginPro 2020, OriginLab Corporation, Northampton, MA, USA).

### Ethics approval

The protocol was reviewed and approved by the Ethical Review Committee of the University of Tsukuba. Informed written consent was obtained from the mothers who donated the placenta after delivery.

## Supplementary Material

Supplementary Figures

Supplementary Tables

Supplementary Table 3
