# Supplementary material for: Microarray analysis of verbenalin-treated human amniotic epithelial cells reveals therapeutic potential for Alzheimer’s Disease
Source: Aging (Albany NY). 2020 Mar 29;12(6):5516–38. doi: 10.18632/aging.102985 (PMC7138585; doi:10.18632/aging.102985)
Supplement: Supplementary Figures [file aging-12-102985-s003..pdf]

SUPPLEMENTARY FIGURE

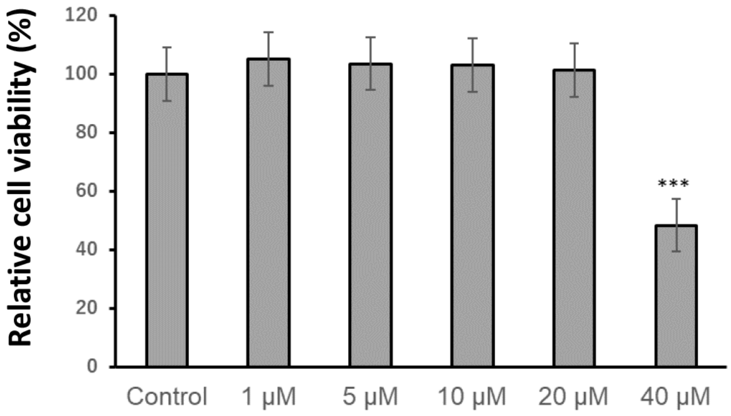

Supplementary Figure 1. hAECs were exposed to verbenalin at concentrations of 1, 5, 10, 20, and 40 μM for 72 h. The control cells were not treated. Cell viability was measured by the MTT assay and was calculated as a percentage of that in the control group (100%). The results are expressed as the means ± standard error of the mean (SEM) of independent experiments (n = 4, 96-well plate). ***p < 0.001 compared with control (untreated) cells.
