# Supplementary material for: Microarray analysis of verbenalin-treated human amniotic epithelial cells reveals therapeutic potential for Alzheimer’s Disease
Source: Aging (Albany NY). 2020 Mar 29;12(6):5516–38. doi: 10.18632/aging.102985 (PMC7138585; doi:10.18632/aging.102985)
Supplement: Supplementary Tables [file aging-12-102985-s002..pdf]

SUPPLEMENTARY TABLES

Please browse Full Text version to see the data of Supplementary Table 3.

Supplementary Table 1. Top 20 upregulated genes (verbenalin-treated hAECs vs D7 Control) and their Gene Ontology (GO).

Gene Symbol	Description	Fold Change (Ver. *vs* D7 Control)	Molecular Function* (GO ID)	Biological Process* (GO ID)
WSB1	WD repeat and SOCS box containing 1	3.07	Protein Binding (GO:0005515)	Protein Ubiquitination (GO:0016567)
SS18#	Synovial sarcoma translocation, chromosome 18	1.98	Protein Binding (GO:0005515); Nuclear Receptor Transcription Coactivator Activity (GO:0030374)	Microtubule Cytoskeleton Organization (GO:0000226); Intracellular Signal Transduction (GO:0035556)
DLEU2	Deleted in lymphocytic leukemia 2 (non-protein coding)	1.97	No Data Available	No Data Available
ASAH2B	N-acyl sphingosine amidohydrolase (non-lysosomal ceramidase) 2B	1.94	N-Acyl sphingosine Amidohydrolase Activity (GO:0017040)	Sphingosine Biosynthetic Process (GO:0046512); Ceramide Catabolic Process (GO:0046514)
TRIM16	Tripartite motif containing 16	1.71	Protein Binding (GO:0005515); Interleukin-1 Binding (GO:0019966)	Histone H3 Acetylation (GO:0043966); Histone H4 Acetylation (GO:0043967)
NCOA7	Nuclear receptor coactivator 7	1.68	Protein Binding (GO:0005515); Nuclear Receptor Transcription Coactivator Activity (GO:0030374)	Negative Regulation of Oxidative Stress-Induced Neuron Death (GO:1903204); Positive Regulation of Transcription By RNA Polymerase II (GO:0045944)
SLC39A8	Solute carrier family 39 (zinc transporter), member 8	1.66	Zinc Ion Transmembrane Transporter Activity (GO:0005385)	Ion Transport (GO:0006811); Transmembrane Transport (GO:0055085)
GTF2H2	General transcription factor IIH, polypeptide 2	1.62	Protein Binding (GO:0005515); Nucleic Acid Binding (GO:0003676)	G Protein-Coupled Receptor Internalization (GO:0002031); DNA Repair (GO:0006281)
NME1	NME/NM23 nucleoside diphosphate kinase 1	1.52	Magnesium Ion Binding (GO:0000287)	GTP (GO:0006183)-, UTP (GO:0006228)-, CTP (GO:0006241)-Biosynthetic Process

TBC1D28	TBC1 domain family, member 28	1.52	GTPase Activator Activity (GO:0005096)	Intracellular Protein Transport (GO:0006886)
GPR89A	G protein-coupled receptor 89A	1.48	Voltage-Gated Ion Channel Activity (GO:0005244)	Ion Transport (GO:0006811); Protein Transport (GO:0015031)
LIMCH1	LIM and calponin homology domains 1	1.46	Protein Binding (GO:0005515); Actin Binding (GO:0003779)	Positive Regulation of Protein Phosphorylation (GO:0001934)

Supplementary Table 2. Top 20 downregulated genes (verbenalin-treated hAECs vs. D7 Control) and their Gene Ontology (GO).

Gene Symbol	Description	Fold Change (Ver. *vs* D7 Control)	Molecular Function* (GO ID)	Biological Process* (GO ID)
CCDC122	Coiled-coil domain containing 122	-2.09	No Data Available	No Data Available
STARD10	StAR-related lipid transfer (START) domain containing 10	-2.09	Protein Binding (Go:0005515); Phospholipid Transporter Activity (Go:0005548)	Phosphatidylcholine Biosynthetic Process (Go:0006656)
CAMK2G#	Calcium/calmodulin-dependent protein kinase II gamma	-2.07	Calmodulin-Dependent Protein Kinase Activity (GO:0004683); Nucleotide Binding (GO:0000166)	Protein Phosphorylation (GO:0006468)
GOLGA2	Golgin A2	-2.02	Protein Binding (Go:0005515); Microtubule Binding (Go:0008017)	Protein Glycosylation (Go:0006486); Microtubule Nucleation (Go:0007020); Golgi Organization (Go:0007030)
NBPF10	Neuroblastoma breakpoint family, member 10;	-2.01	RNA Binding (GO:0003723)	Notch Signaling Pathway (GO:0007219)
PEG10#	Paternally expressed 10	-2.01	Protein Binding (Go:0005515); Rna Binding (Go:0003723); Nucleic Acid Binding (Go:0003676)	Apoptotic Process (Go:0006915)
ARHGAP21#	Rho gtpase activating protein 21	-2	Protein Binding (Go:0005515);	Golgi Organization (Go:0007030); Signal Transduction (Go:0007165)
MAST4	Microtubule associated serine/threonine kinase family member 4	-1.96	Protein Binding (Go:0005515); Magnesium Ion Binding (Go:0000287)	Cytoskeleton Organization (Go:0007010); Protein Phosphorylation (Go:0006468)
FAM65B#	Family with sequence similarity 65, member B	-1.93	No Data Available	No Data Available
TMEM8A#	Transmembrane protein 8A	-1.93	Phospholipase A2 Activity (GO:0004623)	Biological_Process (GO:0008150)
DYNC1H1#	Dynein, cytoplasmic 1, heavy chain 1	-1.89	Nucleotide Binding (GO:0000166); Microtubule Motor Activity (GO:0003777)	Microtubule-Based Movement (GO:0007018); ER To Golgi Vesicle-Mediated Transport (GO:0006888); G2/M Transition of Mitotic Cell Cycle (GO:0000086)

VEGFB#	Vascular endothelial growth factor B	-1.89	Vascular Endothelial Growth Factor Receptor Binding (GO:0005172)	Angiogenesis (GO:0001525); Sprouting Angiogenesis (GO:0002040); Response to Hypoxia (GO:0001666)
SMAP1#	Small arfgap 1	-1.83	GTPase Activator Activity (GO:0005096); Clathrin Binding (GO:0030276)	Positive Regulation of GTPase Activity (GO:0043547)
GOLGB1#	Golgin B1	-1.74	DNA-Binding Transcription Factor Activity (GO:0003700)	ER to Golgi Vesicle-Mediated Transport (GO:0006888)
USF2#	Upstream transcription factor 2, c-fos interacting	-1.65	Protein Homodimerization Activity (GO:0042803)	Regulation of Transcription from RNA Polymerase II Promoter by Glucose (GO:0000430)
CHRNB1	Cholinergic receptor, nicotinic, beta 1 (muscle)	-1.62	Transmembrane Signaling Receptor Activity (GO:0004888)	Postsynaptic Membrane Organization (GO:0001941)
LAMP2	Lysosomal-associated membrane protein 2	-1.61	Protein Domain Specific Binding (GO:0019904)	Protein Targeting (GO:0006605); Autophagy (GO:0006914)
ZKSCAN1	Zinc finger with KRAB and SCAN domains 1	-1.61	DNA-Binding Transcription Factor Activity (GO:0003700)	Regulation of Transcription, DNA-Templated (GO:0006355)
FAM195B	Family with sequence similarity 195, member B	-1.59	No Data Available	No Data Available
PDLIM4#	PDZ and LIM domain 4	-1.59	Protein Binding (Go:0005515); Actin Binding (Go:0003779)	Actin Cytoskeleton Organization (Go:0030036); Excitatory Chemical Synaptic Transmission (Go:0098976)

Supplementary Table 3. List of DEGs in verbenalin-treated hAECs associated with AD.
