# Supplementary material for: Microarray analysis of verbenalin-treated human amniotic epithelial cells reveals therapeutic potential for Alzheimer’s Disease
Source: Aging (Albany NY). 2020 Mar 29;12(6):5516–38. doi: 10.18632/aging.102985 (PMC7138585; doi:10.18632/aging.102985)
Supplement: Supplementary Table 3 [file aging-12-102985-s001..pdf]

Supplementary Table 3. List of DEGs in verbenalin-treated hAECs associated with AD

Gene Symbol	Gene Name	Fold Change	
		Ver. *vs* D0 control	Ver. *vs* D7 control
**Genes up-regulated in brain from patients with Alzheimer's disease (n = 44)**<br><br>**Group A gene list**			
TMEM8A*	Transmembrane Protein 8A	-4.46	-1.93
VEGFB	Vascular Endothelial Growth Factor B	ns	-1.89
GOLGB1	Golgin B1	ns	-1.74
USF2	Upstream Transcription Factor 2, c-Fos Interacting	ns	-1.65
PDLIM4	PDZ And LIM Domain 4	-1.80	-1.59
TAF15§	TAF15 RNA Polymerase II, TATA Box Binding Protein (TBP)-Associated Factor, 68kda	-3.93	-1.56
NR2F6	Nuclear Receptor Subfamily 2, Group F, Member 6	-2.93	-1.54
FAM3A*	Family with Sequence Similarity 3, Member A	-1.66	-1.47
C1S	Complement Component 1, S Subcomponent	3.98	-1.45
EGFR	Epidermal Growth Factor Receptor	-3.35	-1.41
SERTAD2	SERTA Domain Containing 2	1.42	-1.39
ARHGAP17*	Rho GTPase Activating Protein 17	-1.40	-1.38
MYO6	Myosin VI	ns	-1.37
MUC1	Mucin 1, Cell Surface Associated	3.35	-1.34
PAK4	P21 Protein (Cdc42/Rac)-Activated Kinase 4	-1.56	-1.34
TAOK2*	TAO Kinase 2	-1.44	-1.33
TPD52*	Tumor Protein D52	-2.42	-1.32
IQGAP1	IQ Motif Containing GTPase Activating Protein 1	-1.69	-1.31
GNA12	Guanine Nucleotide Binding Protein (G Protein) Alpha 12	-1.73	-1.30
IFNA5*	Interferon, Alpha 5	ns	-1.25
MAP4	Microtubule-Associated Protein 4	-3.75	-1.25
HDGF	Hepatoma-Derived Growth Factor	-1.67	-1.21
LAMP1	Lysosomal-Associated Membrane Protein 1	1.19	-1.20
CUX1§	Cut-Like Homeobox 1	-1.45	-1.18
SEMA4F	Sema Domain, Immunoglobulin Domain (Ig), Transmembrane Domain (TM) And Short Cytoplasmic Domain, (Semaphorin) 4F	1.28	-1.18
SNED1	Sushi, Nidogen and EGF-Like Domains 1	ns	-1.17
TRPS1	Trichorhinophalangeal Syndrome I	ns	-1.17
CPT2*	Carnitine Palmitoyl Transferase 2	ns	-1.15
GOLGA1	Golgin A1	ns	-1.13
NAP1L4	Nucleosome Assembly Protein 1-Like 4	-1.86	-1.12
RPL28	Ribosomal Protein L28	-1.76	-1.11
PZP	Pregnancy-Zone Protein	1.44	-1.10
DLG5	Discs, Large Homolog 5 (Drosophila)	ns	1.10
GOSR2	Golgi SNAP Receptor Complex Member 2	-1.82	1.14
DAZAP2	DAZ Associated Protein 2	ns	1.16
TCF7L1	Transcription Factor 7-Like 1 (T-Cell Specific, HMG-Box)	1.35	1.17
NSUN5P1; NSUN5P2	NOP2/Sun Domain Family, Member 5 Pseudogene 1; NOP2/Sun Domain Family, Member 5 Pseudogene 2	1.48	1.21
EDNRA	Endothelin Receptor Type A	1.14	1.24

ABL1	C-ABL Oncogene 1, Non-Receptor Tyrosine Kinase	-2.17	1.25
DMC1	DNA Meiotic Recombinase 1	-1.07	1.27
TAF11	TAF11 RNA Polymerase II, TATA Box Binding Protein (TBP)-Associated Factor, 28kda	-1.42	1.33
IFIT5	Interferon-Induced Protein with Tetratricopeptide Repeats 5	2.40	1.35
PTTG1IP	Pituitary Tumor-Transforming 1 Interacting Protein	ns	1.40
**Genes down-regulated in brain from patients with Alzheimer's disease (n = 29)**<br>**Group B gene list**			
PEG10[‡]	Paternally Expressed 10	2.77	-2.01
FAM65B	Family with Sequence Similarity 65, Member B	5.44	-1.93
DYNC1H1[†,‡,⊥]	Dynein, Cytoplasmic 1, Heavy Chain 1	ns	-1.89
SMAP1	Small ARFGAP 1	ns	-1.83
MEF2A[§]	Myocyte Enhancer Factor 2a	1.71	-1.52
SLC7A2	Solute Carrier Family 7, Member 2	ns	-1.52
SERGEF	Secretion Regulating Guanine Nucleotide Exchange Factor	ns	-1.45
ARPP19[†]	CAMP-Regulated Phosphoprotein, 19kda	ns	-1.40
PEX19[†]	Peroxisomal Biogenesis Factor 19	ns	-1.35
ZFR[§]	Zinc Finger RNA Binding Protein	1.96	-1.34
OPA1[§]	Optic Atrophy 1 (Autosomal Dominant)	ns	-1.33
STK24[‡,⊥]	Serine/Threonine Kinase 24	1.50	-1.31
GNAS[†,§,⊥]	GNAS Complex Locus	-1.68	-1.27
NDFIP1	Nedd4 Family Interacting Protein 1	-2.36	-1.27
USO1[†]	USO1 Vesicle Transport Factor	-1.19	-1.26
YME1L1[†]	YME1-Like 1 ATPase	1.56	-1.26
LZTS1	Leucine Zipper, Putative Tumor Suppressor 1	ns	-1.25
NFYC	Nuclear Transcription Factor Y, Gamma	-1.81	-1.25
GNB1[†,‡,§]	Guanine Nucleotide Binding Protein (G Protein), Beta Polypeptide 1	-1.99	-1.19
SYNCRIP	Synaptotagmin-binding, Cytoplasmic RNA Interacting Protein	1.69	-1.19
DYNLRB1	Dynein, Light Chain, Roadblock-Type 1	-1.43	-1.18
THY1[†,‡,⊥]	Thy-1 Cell Surface Antigen	-7.97	-1.14
APC[§]	Adenomatous Polyposis Coli	ns	-1.13
RCAN2[†,‡,⊥]	Regulator of Calcineurin 2	1.50	-1.12
SLC12A5[†]	Solute Carrier Family 12 (Potassium/Chloride Transporter), Member 5	-1.36	-1.12
IPO5	Importin 5	-2.30	1.37
EEF1E1	Eukaryotic Translation Elongation Factor 1 Epsilon 1	-1.28	1.33
SLC35A1	Solute Carrier Family 35 (CMP-Sialic Acid Transporter), Member A1	ns	1.20
PMPCB	Peptidase (Mitochondrial Processing) Beta	2.1	1.19
**Interactions of Pathological Hallmark Proteins**<br>**Tubulin Polymerization Promoting Protein/P25, B-Amyloid, And A-Synuclein (n = 22)**			
BDP1	B Double Prime 1, Subunit of RNA Polymerase III Transcription Initiation Factor IIIB	ns	-1.50
PLEKHG6	Pleckstrin Homology Domain-containing, Family G (With RhoGEF Domain) Member 6	1.12	-1.38
SAMD4A	Sterile Alpha Motif Domain Containing 4A	-1.69	-1.38
CTCFL	CCCTC-Binding Factor (Zinc Finger Protein)-Like	1.25	-1.35
SPECC1	Sperm Antigen with Calponin Homology and Coiled-Coil Domains 1	-1.96	-1.21
RNASE1	Ribonuclease, RNAse A Family, 1 (Pancreatic)	ns	-1.17
LIPC	Lipase, Hepatic	ns	-1.14

PZP	Pregnancy-Zone Protein	1.44	-1.10
DLG5	Discs, Large Homolog 5 (Drosophila)	ns	1.10
STON2	Stonin 2	ns	1.11
ADSS	Adenylosuccinate Synthase	1.12	1.13
FOXD2	Forkhead Box D2	ns	1.13
SPNS3	Spinster Homolog 3 (Drosophila)	1.69	1.13
CCR2	Chemokine (C-C Motif) Receptor 2	1.40	1.14
DOCK8	Dedicator of Cytokinesis 8	1.89	1.14
PLEKHS1	Pleckstrin Homology Domain-containing, Family S Member 1	1.26	1.15
ARHGAP11A	Rho GTPase Activating Protein 11A	-3.67	1.17
TBXAS1	Thromboxane A Synthase 1 (Platelet)	1.67	1.18
ANKAR	Ankyrin And Armadillo Repeat Containing	1.39	1.20
SLC35A1	Solute Carrier Family 35 (CMP-Sialic Acid Transporter), Member A1	ns	1.20
EDNRA	Endothelin Receptor Type A	1.14	1.24
SS18	Synovial Sarcoma Translocation, Chromosome 18	-3.31	1.98
**Genes having Strong Association with Late-Onset of Alzheimer Disease (n = 11)**			
ARHGAP19	Rho GTPase Activating Protein 19	ns	-1.11
ARHGAP21	Rho GTPase Activating Protein 21	-1.82	-2.00
CAMK2G	Calcium/Calmodulin-Dependent Protein Kinase II Gamma	-2.81	-2.07
CUBN	Cubilin (Intrinsic Factor-Cobalamin Receptor)	1.46	-1.18
FAM107B	Family with Sequence Similarity 107, Member B	-1.82	-1.35
IFIT5	Interferon-Induced Protein with Tetratricopeptide Repeats 5	2.40	1.35
ITIH5	Inter-Alpha-Trypsin Inhibitor Heavy Chain Family, Member 5	2.87	-1.30
NHLRC2	NHL Repeat Containing 2	ns	-1.22
RNLS	Renalase, FAD-Dependent Amine Oxidase	ns	-1.17
WAPAL	Wings Apart-Like Homolog (Drosophila)	ns	-1.14
YME1L1	YME1-Like 1 ATPase	1.56	-1.26
**Genes Associated with Neurodegeneration (n = 6)**			
CLN3	Ceroid-Lipofuscinosis, Neuronal 3	1.67	1.24
CTSD	Cathepsin D	1.26	-1.51
DYNC1H1	Dynein, Cytoplasmic 1, Heavy Chain 1	ns	-1.89
HSPB1	Heat Shock 27kda Protein 1	ns	-1.31
OPA1	Optic Atrophy 1 (Autosomal Dominant)	ns	-1.33
SBF2	SET Binding Factor 2	ns	-1.32

[*]Genes up-regulated in patients at the incipient stage of AD.

[†]Genes whose expression significantly and positively correlated with the density of CALB1-positive [GeneID=793] GABAergic interneurons in the BA9 brain region across all subjects with psychiatric disorders (n =10).

[‡]Genes whose expression significantly and positively correlated with oligodendrocyte density in layer VI of BA9 brain region (prefrontal cortex) in patients with bipolar disorder (n = 7).

[§]Genes down-regulated in patients at the incipient stage of Alzheimer's disease (n = 6)

[⊥]Genes whose expression was significantly and positively correlated with the number of perineuronal oligodendrocytes in the layer III of BA9 brain region (n = 5).

[§]Genes up-regulated in peripheral lymphocytes from old individuals compared to those from young donors.
